# Field surveys along habitat gradients revealed differences in herpetofauna assemblage in Margalla Hills National Park, Islamabad, Pakistan

**DOI:** 10.3897/BDJ.9.e61541

**Published:** 2021-03-31

**Authors:** Muhammad Rais, Jamal Ahmed, Aiman Naveed, Arooj Batool, Aqsa Shahzad, Razia Bibi, Anum Sajjad

**Affiliations:** 1 Herpetology Lab, Department of Wildlife Management, Arid Agriculture University, Rawalpindi, Pakistan Herpetology Lab, Department of Wildlife Management, Arid Agriculture University Rawalpindi Pakistan; 2 Department of Zoology, Arid Agriculture University, Rawalpindi, Pakistan Department of Zoology, Arid Agriculture University Rawalpindi Pakistan; 3 Department of Biosciences, University of Wah, Rawalpindi, Pakistan Department of Biosciences, University of Wah Rawalpindi Pakistan; 4 Department of Environmental Sciences, International Islamic University, Islamabad, Pakistan Department of Environmental Sciences, International Islamic University Islamabad Pakistan

**Keywords:** abundance, amphibians, diversity, habitat, Margalla Hills, reptiles

## Abstract

This study was conducted to see whether herpetofaunal assemblage differed amongst hiking trails, undisturbed forest and urban areas within the Margalla Hills National Park, Islamabad Capital Territory, Pakistan. Circular plot area-constrained searches (45 plots in each habitat, each plot with an area of 25 m^2^) were used from March 2018 to July 2019. We recorded seven amphibian species, nine lizard species and six snake species. The species richness of amphibians and lizards was the same in the studied strata, while the detection and encounter rate of snakes was lower in the undisturbed forest and urban areas. The encounter rate of amphibians differed significantly between urban areas and hiking trails/undisturbed forest. The encounter rate and population density of lizards differed significantly between undisturbed forest and urban areas. The most frequently encountered amphibian species along the hiking trail and urban areas was *Duttaphrynus stomaticus*, with *Hoplobatrachus tigerinus* in undisturbed forest. The most common and frequently encountered lizard species along the hiking trail and urban areas was *Hemidactylus brookii*, while the *Ophisops jerdonii* was the most frequently seen in undisturbed forest. The most common and frequently encountered snake species along the hiking trail and undisturbed forest was the *Indotyphlops braminus*, while *Ptyas mucosa* was the most common in urban areas. The subsequent bio-assessment, based on herpetofauna, of the Park revealed good to excellent biotic integrity The Park faces threats including livestock grazing, alien invasive vegetation and human disturbance due to settlements, restaurants and tourism-related activities. While several of these threats have been mitigated since the establishment of the Islamabad Wildlife Management Board, the Park still requires improved management, especially regarding regulating tourism.

## Introduction

Many factors, including the geographical location and climatic conditions of a region, determine the diversity and distribution of wildlife species occurring there. Amphibians and reptiles are found in a great variety of habitats (Daniel et al. 2002) ranging from deserts and grasslands, forests and open water and from remote areas to our own houses (Aengals et al. 2011).The species of amphibians and reptiles (herpetofauna) perform a vital role in the ecosystem. They hold immense significance in the food web and as bio-indicators (Iskandar and Erdelen 2006, Stebbins and Cohen 1997).

Protected areas (PAs) are critical to global conservation goals; they are usually created to protect and enhance biodiversity and ecosystem services. Many PAs also contain important features of geological and ecological processes, as well as cultural values (Dudley et al. 2013). Various anthropogenic activities are known to continue to impact habitat and biodiversity, even within these protected areas (Liu et al. 2001, Martinoli et al. 2006). Anthropogenic activities, such as tourism and pollution, are impacting biodiversity of the protected area by influencing presence and absence, as well as dominance status of certain species (Mona et al. 2019). The protected areas in regions with rapid urbanisation may also undergo a significant change due to anthropogenic activities. Further, such areas are likely to experience biotic homogenisation (Angulo et al. 2016).

Article 7 of the Convention on Biological Diversity explicitly requires the identification of components of biological diversity and their monitoring through sampling and other appropriate techniques. Since complete documentation of biodiversity seems impossible, recognition of some elements of biodiversity and their monitoring may be achieved. Hence, identification of understudied wildlife species or group of species may aid in the biodiversity conservation of the areas (Burley 1998). Monitoring of biodiversity helps evaluate outcomes of conservation actions and testing the success of different types of protected areas. Various monitoring methods, such as pugmarks and aerial surveys for African elephants, apes and ungulates (Starkey et al. 2014) and time-constrained searches (Visual Encounter Surveys), area-constrained searches, bio-acoustics, pitfall trapping and egg mass surveys for herpetofauna have widely been used (Campbell and Christman 1982, Corn and Bury 1990, Crump and Scott Jr 1994, Reynolds et al. 1997, Zimmerman 1994). Karr and Dudley (1981) defined biotic integrity as "the ability of an ecosystem to support and maintain a balanced, integrated, adaptive community of organisms having a species composition, diversity, and functional organization comparable to that of the natural habitats within a region”. Karr et al. (1986) explained that the ‘strength of the index of biotic integrity (IBI) is its ability to integrate information from individual, population, community, zoogeographic and ecosystem levels into a single ecologically based index’. The IBI and its variations have subsequently been used in a variety of aquatic habitats (Butcher et al. 2003, Simon et al. 2000) and in a modified form in terrestrial environments using invertebrates (Bisevac and Majer 1999), birds (Bradford et al. 1998, O’Connell et al. 1998, Glennon and Porter 2005) and amphibians (Simon et al. 2000), but never reptiles. However, Thompson et al. (2008) developed a rehabilitation and degradation index (RDI) to quantify reptile’s rehabilitation success for terrestrial habitats (mine site waste dumps and adjacent undisturbed areas). The National Park Service, USA, has used fish to develop the IBI for the Great Smoky Mountains National Park, North Carolina and Tennessee, USA (https://irma.nps.gov/DataStore/Reference/Profile/2238679), while Milner et al. (2006) used macroinvertebrate communities for Denali National Park, Alaska, USA. These authors have defined, used and established different metrics. Andreasen et al. (2001) proposed that the terrestrial index of ecosystem integrity (TIEI) should be multi-scale, grounded in natural history, flexible and measurable. No attempt has been made to develop such baseline scale for any national park of Pakistan.

Pakistan has varied topography and bioclimatic conditions which are reflected in the diversity of ecological zones and wildlife (Roberts 1991, Roberts 1992, Roberts 1997). The main protected areas of Pakistan include National Parks, Wildlife Sanctuaries, Game Reserves and Community Controlled Hunting Areas (GOP (Government of Pakistan) 2015). IUCN (2000) suggested the establishment of new protected areas, improvement of standards and reclassification of protected areas in Pakistan, based on biodiversity richness, ecosystem functioning, uniqueness and scenic/recreational significance. To date, about 34 National Parks have been established in the country. Anwar (2020) identified the lack of scientific studies and data in the protected areas as one of the major constraints in the management of protected areas. The herpetofauna species inventory of Margalla Hills National Park is available (Masroor 2011). However, the study did not provide data on abundance or information on comparison of herpetofauna across different habitats/land uses of the National Park. Since the area enjoys legal protection as a National Park, we attempted to establish if the diversity and abundance of herpetofauna differed along hiking trails, undisturbed forest and urban areas of the Park. We aimed to provide data on herpetofaunal abundance and create an index for monitoring and bio-assessment of the National Park. The Park faces threats, such as human disturbance due to settlements, restaurants and tourism-related activities, livestock grazing, encroachment and spread of invasive vegetation, such as *Lanatana camara* (Anon 2007). Our data on abundance and encounter rate could be used as a basis to evaluate conservation status and monitor populations of herpetofauna in the National Park. Likewise, the index of biotic integrity may serve as a scale to examine the ecological health of the Park in the future.

## Materials and methods

We conducted the present study in Margalla Hills National Park (MHNP) (33.7481°N, 73.0051°E), Islamabad Capital Territory (ICT), Pakistan. The Park is located at an elevation of 1,604 m above sea level, at the north-eastern side of Islamabad Capital Territory. It spreads over an area of 15,880 ha including Margalla Hills (12,802 ha), Shaker Parian (1376 ha) and Rawal Lake (1702 ha) (Anwar and Chapman 2000). The Park has a rough topography with steep slopes and is predominantly limestone rock (Shinwari and Khan 2001). The region has a subtropical, semi-arid climate and lies within the monsoon belt, resulting in two rainy seasons: January-March winter rains and July-September summer rains. The mean annual precipitation is 1,000 mm, while the range of minimum and maximum annual temperature is 1–15°C and 20–40°C, respectively (Anon 2007). The Park features sub-tropical broad-leaf evergreen forest (SBEF) dominated by scrub vegetation, such as *Acacia modesta*, *Olea ferruginea*, *Maytenus royleanus*, *Carissa apaca*, *Dodonea viscosa*, *Clematis grata*, *Oplismenus burmanii* and *Cyanodon dactylon* (Shinwari and Khan 2001). The Park was selected for the present study because it lacks data on abundance of herpetofauna. The Park is easy to access and our research was conducted under a research grant identified in the funding programme section.

### Study Design

**Figure 1.**
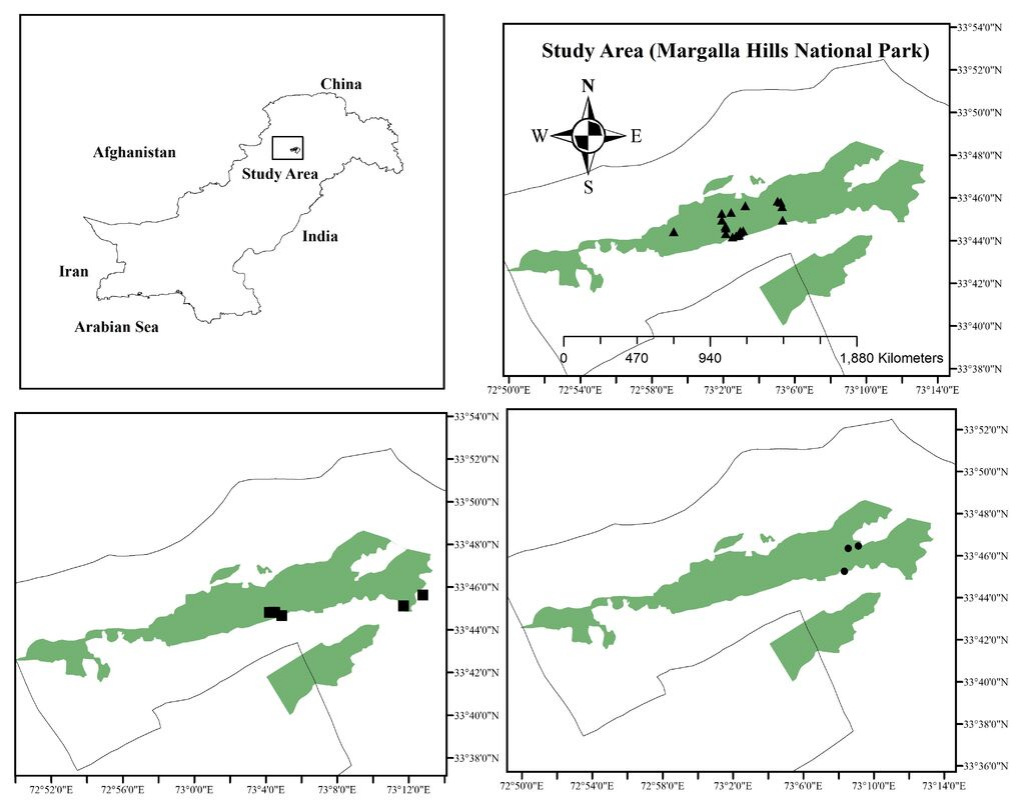
Map of Margalla Hills National Park, Islamabad, Pakistan, showing locations of the sampling sites along hiking trails (top right, sites denoted as triangles), undisturbed forest (bottom left, squares) and urban area (bottom right, circles)

We recorded the data from March 2018 to July 2019 through a total of 42 surveys (field days) excluding winter months (Decemeber-February). We surveyed in early morning (two hours after sunrise), afternoon (12:00 to 14:00 h) and evening/nocturnal (two hours after sunset). We selected three major habitat types inside the National Park. The hiking trails (number of sampling sites = 19) which experience moderate to high level of tourist activity mostly hiking, sightseeing, bird watching and recreational visits. The undisturbed forest area (n = 18) was characterised by low or no human disturbance and urban areas (n = 16) with high level of human disturbance, road network, traffic and restaurants (Fig. 1, Suppl. material 1). The nature and extent of human disturbance differed between hiking trails and urban settlements. The former is limited to outdoor recreation by the tourists, noise and music, while the latter by high level of disturbance, such as construction, traffic and solid waste disposal.

We used area-constrained searches (Greater et al. 2008) and employed circular plot searching. Each circular plot was surveyed once and had a radius of 5 m, measured using a rope. We haphazardly set out 135 searching plots in total, with 45 plots in each habitat (ranging from 1 to 4 plots around 400-500 m of the sampling site). Each plot had an area of 25 m^2^ or 0.0025 ha, resulting in a total area sampled of 0.3375 ha (0.1125 ha/habitat). All potential refuges within the circular plot (rocks, stones, vegetation, fallen logs, tree bark and cavities) were searched. The adult amphibians, tadpoles, small lizards and blind snakes were hand-picked or sometimes collected by using dip nets and were later released in the same plot. We followed Khan (2006) for species identification.

### Data Analysis

The data on abundance were subjected to basic statistics (mean ± standard error). We spent > 250 field hours, but retained 215 hrs, during which we gathered data, for the calculation of encounter rate. To standardise the effort and for future replication, we calculated the encounter rate (ER) as number of individuals/observation time (observation time: total field hours (215)/6 hours per field day = 36 hours) and population density (PD) as number of individuals/area (ha). We subjected the data (encounter rate and population density) to a normality test (Shapiro-Wilk Test) and log transformed the non-normal data (ER and PD of amphibians in urban areas). We used one-way ANOVA (α = 0.05) to compare the means (ER, PD) amongst the three studied habitats and Tukey's Test to make pair-wise comparison. The data for snakes (ER and PD) was non-normal and tranformation did not help achieve the normality. We, therefore, used the Krsukal Wallis Test (α = 0.05). The analysis was done using QED Statistics, Version 1.1 (Henderson and Seaby 2007). The information whether the species was a habitat generalist (score 5) or specialist (10) was obtained from Khan (2006); the conservation status was evaluated (5) or not-evaluated (10) from the IUCN Red List of Threatened Species (2020) and whether the species was widely distributed in Pakistan and invasive in elsewhere in the world (score 5) or not (10) from Amphibian Web Database 2020, Reptile-database 2020. This was added with data on encounter rates gathered during the present study to determine whether the species was uncommon (encounter rate 0.10-0.30, score 20), frequent (0.31-0.50, 15), common (0.51-0.80, 10) or abundant (0.81 and above, 5) in order to develop the index of biotic integrity (IBI). We then added scores for each species at each habitat to produce a total score which was then assigned a condition category. The maximum possible IBI score was 1000 and thus we rated 900-1000 as excellent biological integrity, 500-800 good biotic integrity and < 800 as poor biotic integrity.

## Results

We recorded 302 individuals of seven amphibian species, 303 individuals of nine lizard species and 32 individuals of six snake species from the National Park (Suppl. material 3). We recorded 71 amphibians of seven different species, 103 lizards of nine different species and 13 snakes of six different species from the hiking trails. From the undisturbed forests, we recorded 142 amphibians of seven different species, 145 lizards of nine different species and 16 snakes of three different species. Finally, in the urban areas in and around the Park, we recorded 89 amphibians of seven different species, 303 lizards of nine different species and 32 snakes of two different species (Suppl. material 3). The most common and frequently encountered amphibian species in the Park included the Indus Valley Toad (*Duttaphrynus stomaticus*) and Bull Frog (*Hoplobatrachus tigerinus*). We found the Spotted Barn Gecko (*Hemidactylus brookii*) and Rat Snake (*Ptyas mucosa*) as the most common and frequently encountered lizard and snake species, respectively.

**Figure 2.**
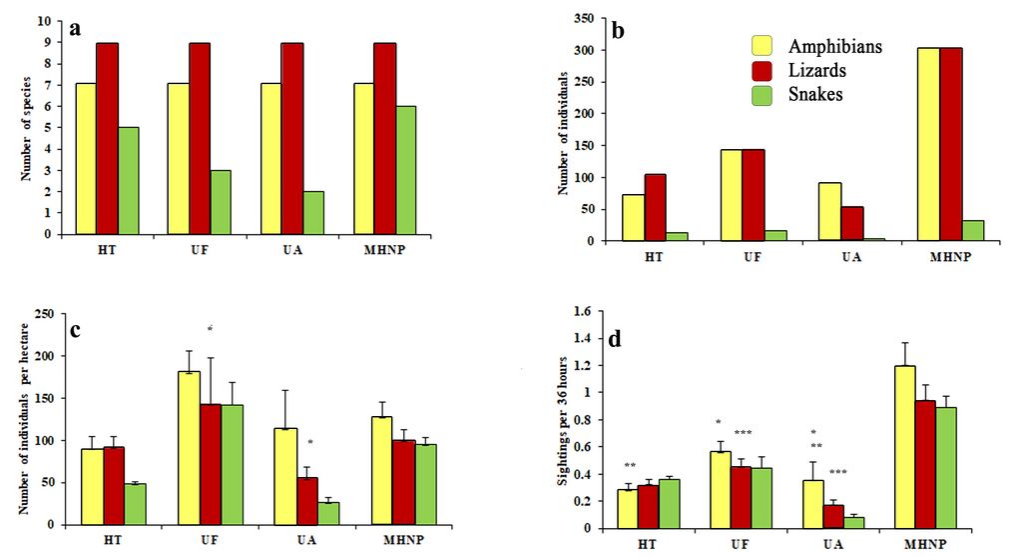
Number of species (a) number of individuals; (b) population density; (c) as number of individuals per ha and encounter rate; (d) as sightings per 36 hours of amphibians and reptiles recorded from hiking trails (HT), undisturbed forest (UF) and urban area (UA) of Margalla Hills National Park (MHNP), Islamabad, Pakistan. Similar symbol (*/**/***) over a bar within the same graph shows statistically significant different values (P < 0.05).

The species richness of amphibians and lizards was the same across the studied strata, while the detection and encounter rate of snakes was low in undisturbed forest and urban areas. The encounter rate of amphibians differed significantly amongst the studied habitats (F_2, 18_ = 32.07 P < 0.05), while the encounter rate (F_2, 24_ = 8.59, P < 0.05) and population density (F_2, 24_ = 8.58, P < 0.05) of lizards differed significantly (Suppl. material 2, Fig. 2). The most frequently encountered amphibian species along the hiking trail and urban areas was *D. stomaticus*, with *H. tigerinus* in undisturbed forest (Suppl. material 3). The most common and frequently encountered lizard species along the hiking trail and urban areas was *H. brookii*, while the Rugose Spectacled Lacerta (*Ophisops jerdonii*) was the most frequently seen in undisturbed forest. The most common and frequently encountered snake species along the hiking trail and undisturbed forest was the Blind Snake (*Indotyphlops braminus*), while Rat Snakes, (*Ptyas mucosa*), were the most common in urban areas. The subsequent bio-assessment, based on herpetofauna, of the Park revealed good to excellent biotic integrity (Suppl. material 3).

## Discussion

This study recorded seven species of amphibian and 15 of reptiles from the National Park. The most common and frequently encountered amphibians of the Park were *D. stomaticus* and *H. tigerinus*, of lizards, it was *H. brookii* and of snakes, it was *P. mucosa*. Rais et al. (2015) recorded five species of amphibians and 22 of reptiles from Rawalpindi and Islamabad. Masroor (2011) recorded 41 species (which included nine amphibian and 32 reptilian species) during a seven-year study at MHNP, Islamabad. Masroor (2011) recorded the Marbled Balloon Frog (*Uperodon systoma*) only found from sub-tropical semi-evergreen forest, while the generalist lizard species, Oriental Garden Lizard (*Calotes versicolor*), was the most abundant species recorded from almost all types of habitats within the Park. We documented fewer species than Masroor (2011). One reason for fewer numbers of species in our study was the exclusion of the wetland (Rawal Lake) from MHNP which resulted in three testudine species (*Pangshura smithii smithii*, *Nilssonia gangeticus* and *Lissemys punctata andersoni*) being excluded, while some parts of the Park could not be visited due to security issues. Another important reason could be the effect of detection probabilities which greatly influence population dynamics and demographic parameters. Imperfect detection led to discrepancies in return rates and survival probability estimates of the Torrent Frog (*Hylodes asperi*) (Guimares et al. 2014). Although we assumed that the detection was perfect and remained constant during our study, we cannot be certain about this in previous studies.

The present study reports a significant difference in abundance of herpetofauna amongst studied trails, undisturbed forest and urban areas. We attribute more sightings along the hiking trails due to better visibility which leads to higher detection rates. There is a dearth of information on variation in the detection due to vegetation. However, Ryan et al. (2002) reported that communities of amphibians and reptiles varied amongst three different terrestrial habitats (recent clearcut, pine plantation and mixed pine–hardwood forest) in Woodbury Tract, South Carolina, USA.

Attempts have been made to assess the biotic integrity of habitats, based on phytoplankton (Al-Janabi 2016), invertebrates (Deshon 1995) and fish (Minns et al. 1994, Drake and Pereira 2002), but seldom using amphibians (Simon et al. 2000). Nonetheless, amphibians have long been used as bio-indicators in many parts of the world. Although species richness (amphibians and lizards) did not change across habitats, perceived abundance did. Some species were more abundant at one habitat. For instance, *Duttaphrynus melanostictus* was more abundant in urban areas, while *Microhyla nilphameriensis* and *Hoplobatrachus tigerinus* were more abundant in undisturbed forest areas showing their association and adaptation to natural and anthropogenic settings.

The current study for the first time presented data on the encounter rate of herpetofauna which could be used for monitoring and comparing future management of the Park. Likewise, a first index of bio-assessment of the Park has been created and presented. Most of the threats, such as human disturbance, grazing, encroachment and invasive species, have recently been mitigated, after the establishment of Islamabad Wildlife Management Board. Although many of the illegal small villages have been vacated and encroached land recovered, the Park still requires improved management, especially relating to tourism regulation and other human impacts.

## Supplementary Material

DE197DFD-9B15-5684-B56A-30A7627A0F7C 10.3897/BDJ.9.e61541.suppl1 Supplementary material 1 Geographical coordinates and plot details Data type Coordinates File: oo_480141.xls https://binary.pensoft.net/file/480141 Muhammad Rais, Jamal Ahmed, Aiman Naveed, Arooj Batool, Aqsa Shahzad, Razia Bibi and Anum Sajjad

19072401-91BF-559D-A3EE-6CA816AD2205 10.3897/BDJ.9.e61541.suppl2 Supplementary material 2 ANOVA and Kruskal-Wallis Test Output Data type Statistics File: oo_516350.xls https://binary.pensoft.net/file/516350 Muhammad Rais, Jamal Ahmed, Aiman Naveed, Arooj Batool, Aqsa Shahzad, Razia Bibi and Anum Sajjad

2AEDB89E-FA5E-58BE-A2A3-FFEF4666B1CD 10.3897/BDJ.9.e61541.suppl3 Supplementary material 3 Species list Data type Occurrences Brief description Number of individuals (N), population density (PD), encounter rate (ER) and Index of biotic integrity (IBI) for the studied habitats (hiking trails HT, undisturbed forest UF and urban areas UA) of Margalla Hills National Park (MHNP), Islamabad Capital Territory, Pakistan. Index of biotic integrity scoring criteria (IBI): Habitat (H), Conservation status (CS), Widely distributed in Pakistan and reported as Invasive elsewhere in the world (I); Habitat generalist (score 5) or specialist (10), conservation status evaluated (5) or not-evaluated (10), whether the species was invasive (score 5) or not (10), whether the species was uncommon (encounter rate 0.10-0.30, score 20), frequent (0.31-0.50, 15), common (0.51-0.80, 10) or abundant (0.81 and above, 5). IBI score: 900-1000 represented excellent (E) biological integrity, 700-900 good (G) biotic integrity and < 700 poor biotic integrity File: oo_480162.xlsx https://binary.pensoft.net/file/480162 Muhammad Rais, Jamal Ahmed, Aiman Naveed, Arooj Batool, Aqsa Shahzad, Razia Bibi Anum Sajjad

## References

[B6072721] Aengals R, Kumar VS, Palot MJ Updated checklist of Indian reptiles. Zoological Survey of India. https://zsi.gov.in/checklist/Reptiles.

[B6072737] Al-Janabi ZZ (2016). Use of Phytoplankton Index of Biological Integrity (P-IBI) as a tool to evaluate Tigris River Health. Mesopotamia Environmental Journal.

[B6072746] Database Amphibian Web https://amphibiaweb.org/.

[B6792556] Andreasen J. K., ONeill R. V., Noss R., Slosser N. C. (2001). Considerations for the development of a terrestrial index of ecological integrity. Ecological Indicators.

[B6393453] Angulo E, Boulay R, Ruano F, Tinaut A, Cerdá X (2016). Anthropogenic impacts in protected areas: Assessing the efficiency of conservation efforts using Mediterranean plant communities. PeerJ.

[B6072729] Anon (2007). Margalla Hills National Park ecological baseline draft report (D7BL1MHP). HWF and Capital Development Authority..

[B6072754] Anwar M, Chapman A (2000). Feeding habits and food of Grey Goral in the Margalla Hills National Park. Pakistan Journal of Agricultural Research.

[B6393591] Anwar M (2020). Review of protected areas system of Pakistan to include new categories as provided by IUCN, Green Pakistan Program.

[B6393519] Bisevac L, Majer J. D, Ponder W. F, Lunney D (1999). An evaluation of invertebrates for use as success indicators for minesite rehabilitation. The other 99%: The conservation and biodiversity of invertebrates.

[B6393532] Bradford D. F, Franson S. E, Neale A. C, Heggem D. T, Miller G. R, Canterbury G. E (1998). Bird species assemblages as indicators of biological integrity in Great Basin Rangeland. Environmental Monitoring and Assessment.

[B6393471] Burley F. W, Council National Research (1998). Monitoring biological diversity for setting priorities in conservation. Biodiversity.

[B6393510] Butcher J. T, Stewart P. M, Simon T. P (2003). A benthic community index for streams in the northern lakes and forests ecoregion. Ecological Indicators.

[B6792709] Campbell H. W, Christman S. P, Scott Jr N. J (1982). Field Techniques for Herpetofaunal Community Analysis. Herpetological Communities, Wildlife Research Report 13, U.S.

[B6792685] Corn P. S, Bury R. B (1990). Sampling Methods for Terrestrial Amphibians and Reptiles, General Technical Report, U.S. Department of Agriculture, Forest Service, PNW-GTR-256, Portland, Oregon..

[B6792641] Crump M. L, Scott Jr N., Heyer W. R., Donnelly M. A., McDiarmid R. W., Hayek L. C., Foster M. S. (1994). Visual Encounter Surveys Measuring and Monitoring Biological Diversity: Standard Methods for Amphibians. Measuring and Monitoring Biological Diversity: Standard Methods for Amphibian.

[B6072764] Daniel K, Hirshleifer D, Teoh S. H (2002). Investor psychology in capital markets: Evidence and policy implications. Journal of Monetary Economics.

[B6072773] Deshon J. E, Davis W. S, Simon T. P (1995). Development and application of the Invertebrate Community Index (ICI). Biological assessment and criteria: Tools for water resource planning and decision making.

[B6072786] Drake M. T, Pereira D. L (2002). Development of a fish-based index of biotic integrity for small inland lakes in central Minnesota. North American Journal of Fisheries Management.

[B6072795] Dudley N, Shadie P, Stolton S (2013). Guidelines for applying protected area management categories including IUCN, WCPA best practice guidance on recognizing protected areas and assigning management categories and governance types. Best practice protected area guidelines series. IUCN.

[B6393551] Glennon M. J, Porter W. F (2005). Effects of land use management on biotic integrity: An investigation of bird communities. Biological Conservation.

[B6072821] Pakistan) GOP (Government of (2015). Pakistan National Biodiversity Strategy and Action Plan..

[B6072839] Greater G, Buhlmann K, Wilkinson L, Gibbons J. W (2008). Inventory and monitoring: recommended techniques for reptiles and amphibians, with application to the US and Canada..

[B6792578] Guimares M, Doherty P. F., Mungua-Steyer R (2014). Strengthening Population Inference in Herpetofaunal Studies by Addressing Detection Probability. South American Journal of Herpetology.

[B6793573] Henderson P. A, Seaby R. H.M (2007). QED Statistics. www.pisces-conservation.com.

[B6072866] Iskandar D. T, Erdelen W. R. (2006). Conservation of amphibians and reptiles in Indonesia: issues and problems. Amphibian and Reptile Conservation.

[B6393582] IUCN (2000). Pakistan protected area system review and action plan.

[B6072886] Species IUCN Red List of Threatened https://www.iucnredlist.org.

[B6393492] Karr J. R, Dudley D. R (1981). Ecological perspective on water quality goals. Environmental Management.

[B6393501] Karr J. R, Fausch F. D, Angermeiser P. L, Yant P. R, Schlosser I. J (1986). Assessing biological integrity in running waters: A method and its rationale.

[B6072996] Khan M. S (2006). Amphibians and Reptiles of Pakistan.

[B6073004] Liu J, Linderman M,, Ouyang Z, An L, Yang J, Zhang H (2001). Ecological degradation in protected areas: The case of Wolong Nature Reserve for Giant Pandas. Science.

[B6073056] Martinoli A, Preatoni Galanti D. V, Codipietro P, Kilewo M, Fernandes C. A.R, Wauters L. A, Tosi G (2006). Species richness and habitat use of small carnivores in the Arusha National Park (Tanzania).. Biodiversity Conservation 15:.

[B6073101] Masroor R (2011). An annotated checklist of amphibians and reptiles of Margalla Hills National Park, Pakistan.. Pakistan Journal of Zoology.

[B6792613] Milner A. M, Conn S. C., Brown L. E (2006). Persistence and stability of macroinvertebrate communities in streams of Denali National Park, Alaska: implications for biological monitoring. Fresh Water Biology.

[B6073112] Minns C. K, Cairns V. W, Randall R. G, Moore J. E (1994). An Index of Biotic Integrity (IBI) for fish assemblages in the littoral zone of Great Lakes' areas of concern. Canadian Journal of Fisheries and Aquatic Sciences.

[B6393442] Mona M. H, El-Naggarb H. A, El-Gayara E. E, Masood M. F, Mohamed E. S.N (2019). Effect of human activities on biodiversity in Nabq Protected Area, South Sinai, Egypt. Egyptian Journal of Aquatic Research.

[B6393543] O’Connell T. J, Jackson L. E, Brooks R. P (1998). The Bird Community Index: A tool for assessing biotic integrity in the Mid-Atlantic Highlands.

[B6073209] Rais M, Akram A, Ali S. M, Asadi M. A, Jahangir M, Jilani M. J, Anwar M (2015). Qualitative analysis of factors influencing the diversity and spatial distribution of herpetofauna in Chakwal tehsil (Chakwal District), Punjab, Pakistan. Herpetological Conservation and Biology.

[B6073229] Reptile-database https://www.reptile-database.com/..

[B6792694] Reynolds R., Fritts T., Gotte S., Icochea J., Tello G., Dallmeier F., Alonso A. (1997). Amphibians and Reptiles I. Biodiversity Assessment and Long-term Monitoring of the Lower Urubamba Region, Peru: San Martin-3 and Cashiriari-2 Well Sites, SI/MAB Series #1. Smithsonian Institution/MAB Biodiversity Program.

[B6073245] Roberts T. J (1991). Birds of Pakistan.

[B6073253] Roberts T. J (1992). Birds of Pakistan..

[B6073237] Roberts T. J (1997). Mammals of Pakistan..

[B6792566] Ryan T. J., Philippi T., Leiden Y. A., Dorcas M. E., Wigley T. B., Gibbons J. W. (2002). Monitoring herpetofauna in a managed forest landscape: effects of habitat types and census techniques. Forest Ecology and Management.

[B6073261] Shinwari M. I, Khan M. A (2001). Marketable medicinal plants of Margalla Hills National Park, Islamabad,Pakistan. Pakistan Journal of Forestry (Pakistan).

[B6073270] Simon T. P, Jankowski R, Morris C (2000). Modification of an index of biotic integrity for assessing vernal ponds and small palustrine wetlands using fish, Crayfish, and amphibian assemblages long Southern Lake Michigan.. Aquatic Ecosystem Health and Management.

[B6393484] Starkey M, Scholtz O, Taylor G (2014). Wildlife monitoring practices and use in Central Africa.

[B6073279] Stebbins R. C, Cohen N. W (1997). A natural history of amphibians.

[B6792627] Thompson S. A., Thompson G. G., Withers P. C. (2008). Rehabilitation index for evaluating restoration of terrestrial ecosystems using the reptile assemblage as the bio-indicator. Ecol Indic.

[B6792725] Zimmerman B. L., Heyer W. R., Donnelly M. A., McDiarmid R. W., Hayek L. C., Foster M. S. (1994). Audio Strip Transects. Measuring and Monitoring Biological Diversity: Standard Methods for Amphibians.

